# Comparative efficacy and safety of abrocitinib, baricitinib, and upadacitinib for moderate‐to‐severe atopic dermatitis: A network meta‐analysis

**DOI:** 10.1111/dth.15636

**Published:** 2022-07-27

**Authors:** Huiying Wan, Haiping Jia, Tian Xia, Dingding Zhang

**Affiliations:** ^1^ Department of Dermatology, Sichuan Provincial People's Hospital University of Electronic Science and Technology of China Chengdu China; ^2^ Department of Immunology and Microbiology, North Sichuan Medical College Nanchong China; ^3^ Department of Pathology, The 452nd Hospital of People's Liberation Army Chengdu China; ^4^ Sichuan Provincial Key Laboratory for Genetic Disease, Sichuan Provincial People's Hospital University of Electronic Science and Technology of China Chengdu China

**Keywords:** abrocitinib, atopic dermatitis, baricitinib, Janus kinase inhibitor, network meta‐analysis, upadacitinib

## Abstract

Janus kinase (JAK) inhibitors have become promising treatments for atopic dermatitis (AD), however no study directly comparing JAK inhibitors with each other has been reported. We conducted this network meta‐analysis to determine the comparative efficacy and safety of three common oral JAK inhibitors including abrocitinib, baricitinib, and upadacitinib for moderate‐to‐severe AD. We first identified eligible studies from published meta‐analyzes, then we searched PubMed to obtain additional studies published between February and July 2021. Clinical efficacy and safety were evaluated as primary and secondary outcome, respectively. After extracting data and assessing methodological quality, we utilized ADDIS 1.4 software to conduct pair‐wise and network meta‐analyzes. Ten eligible studies were included in the final analysis. Pooled results that abrocitinib, baricitinib, and upadacitinib obtained higher investigator global assessment (IGA), eczema area, and severity index (EASI) response, however abrocitinib and upadacitinib caused more treatment‐emergent adverse events (TEAEs) regardless of doses, compared with placebo. Network meta‐analyzes revealed that upadacitinib 30 mg was superior to all regimens and upadacitinib 15 mg was better than remaining regimens except for abrocitinib 200 mg in terms of IGA and EASI response. Moreover, abrocitinib 200 mg was superior to abrocitinib 100 mg, baricitinib 1 mg, 2 mg, and 4 mg for clinical efficacy. However, upadacitinib 30 mg caused more TEAEs. Abrocitinib, baricitinib, and upadacitinib were consistently effective therapies in adult and adolescent patients with AD; however, upadacitinib 30 mg may be the optimal option in short‐term studies. More efforts should be done to reduce the risk of TEAEs caused by upadacitinib 30 mg.

## 1 INTRODUCTION

Atopic dermatitis (AD) is a chronic recurring inflammatory skin disorder, which is characterized by intense pruritus and eczematous lesions.^1^, ^2^ It's estimated that approximately 20% of children and 10% of adults experience this condition around the world.^3^, ^4^ AD has been found to be associated with increased risk of psychological distress,^5^ poor quality of life (QoL),^6^, ^7^ impaired work‐related performance,^6^, ^8^ and increased healthcare expenditure.^9^, ^10^ Therefore, it's imperative to effectively treat AD. Unfortunately, it's still a challenge for the diagnosis and treatment of AD due to its various presentations.^11^

The etiology and mechanisms of AD has not yet been clarified,^12^ and current treatments were applied to mainly reduce the severity and frequency of symptoms^13^ and establish long‐term disease control.^14^ Although topical interventions remain the mainstay option of AD, systemic therapy has also been recommended for patients with inadequate treatment response^15^, ^16^, ^17^ owing to an improved understanding of the molecular mechanism of AD.^18^ It's exciting that Janus kinase signal transducers and activators of transcription (JAK–STAT) pathway has been found to play a critically important role in the development and progression of AD,^19^ and thus researchers and practitioners focused their attention on JAK inhibitors.^20^

At present, a series of clinical trials have revealed that several JAK inhibitors including oral and topical administration have the potential of significantly improving the clinical outcomes of AD patients with inadequate responses to conventional treatment regimes.^21^, ^22^ Meanwhile, some meta‐analyzes further established the treatment efficacy of JAK inhibitors in treating AD.^23^, ^24^, ^25^ In theory, different kinds of JAK inhibitors should have different effects for the treatment of AD,^25^ however there were no head‐to‐head comparisons between JAK inhibitors. We therefore conducted this network meta‐analysis to provide an up‐to‐date synthesis of the comparative efficacy and safety of three common oral JAK inhibitors including abrocitinib, baricitinib and upadacitinib for moderate‐to‐severe AD as monotherapy.

## 2 MATERIALS AND METHODS

This network meta‐analysis was conducted in accordance with the preferred reporting items for systematic reviews and meta‐analysis (PRISMA) for network meta‐analysis (PRISMA‐NMA)^26^, ^27^ and the Cochrane collaboration (CC).^28^ No ethical approval and informed consent were required because of this was a network meta‐analysis of published studies.

### 2.1 Search strategy

We first captured published meta‐analyzes from PubMed, and then we conducted an updated search in PubMed to identify additional studies published between February and July 2021 according to the latest time in published meta‐analyzes. Two independent reviewers conducted the literature search. Details of search query for PubMed were summarized in Table S1. In the event of any disagreement, a third senior reviewer contributed to the discussions.

### 2.2 Study selection

We first imported records from PubMed and published meta‐analyzes into EndNote software, and then removed duplicates. Next step, we removed irrelevant records through screening the titles and abstracts of records. Finally, we accessed the full text of all potentially eligible studies and determined eligible studies through checking eligibility. Two independent reviewers conducted study selection in order to avoid selection bias. In the event of any disagreement, a third senior reviewer contributed to the discussions.

### 2.3 Selection criteria

We only included randomized controlled trials (RCTs) in this network meta‐analysis in order to avoid potential selection and confounding bias.^29^ RCTs comparing abrocitinib, baricitinib, or upadacitinib against placebo were included without limitations on sex, ethnicity, or treatment duration. We excluded studies with insufficient information and combination therapies, and enrolling pediatric participants. Moreover, studies with ineligible design such as comments, narrative review, and conference abstracts were also excluded.

### 2.4 Outcomes

According to a previous meta‐analysis, the efficacy outcomes^30^ included (a) a ≥ 75% decrease in eczema area and severity index (EASI) from baseline (defined as EASI‐75 response) and (b) an investigator's global assessment (IGA) score of 0 (clear) or 1 (almost clear) with a ≥ 2‐point reduction from baseline (defined as IGA response). The safety outcome was defined as the development of treatment‐emergent adverse events (TEAEs).^30^

### 2.5 Data extraction

Two independent reviewers conducted data extraction. The following data were extracted from eligible studies including: general information (first author, publication year, and study design), patient characteristics (sample size, mean age, definition criteria, and AD severity), details of the targeted JAK inhibitors (administration route, dosage, frequency, and endpoint), and outcomes (efficacy and safety). Details of the risk of bias were also extracted to assess the methodological quality. In the event of any disagreement, a third senior reviewer contributed to the discussions.

### 2.6 Assessment of risk of bias

Two independent reviewers assessed the risk of bias with the CC risk of bias assessment tool.^31^ In this tool, seven items including random sequence generation, allocation concealment, blinding of personnel, participants, and outcome assessors, incomplete data, selective reporting, and other sources were assessed as having a low, high, or unclear risk of bias. In the event of any disagreement, a third senior reviewer contributed to the discussions.

### 2.7 Statistical analysis

We utilized the aggregate data drug information system (ADDIS) software (Groningen, the Netherlands, www.drugis.org) to conduct all statistical analyzes, and network meta‐analysis was conducted based on Markov Chain Monte Carlo (MCMC) simulation. The following parameters were used to estimate results of network meta‐analysis including 4 chains, 20,000 tuning and 50,000 simulation iterations, thinning interval of 10, 10,000 inference samples, and variance scaling factor of 2.5.^32^ The Brooks Gelman–Rubin statistical method was utilized to evaluate the convergence of data. Data achieved good convergence if a potential proportional reduction factor (PRF) was close to 1.^33^, ^34^ Efficacy and safety outcomes in this network meta‐analysis were dichotomous variables, and thus OR with 95% confidence interval (CI) or creditable interval (CrI) were used to express the results.

### 2.8 Heterogeneity and inconsistency test

In this network meta‐analysis, heterogeneity across studies was first qualitatively examined using the Cochrane Q test,^35^ and then I^2^ statistic was utilized to quantify the level of heterogeneity.^36^ It's noted that the structure of all comparisons was simple star‐shaped and no closed‐loop was available. It's therefore impossible to test whether the presence of inconsistency or not.^37^, ^38^

### 2.9 Subgroup analysis

For three JAK inhibitors considered in this network meta‐analysis, different doses were utilized in eligible studies. We therefore conducted further subgroup network meta‐analysis to investigate the comparative efficacy and safety of the most common doses of target JAK inhibitors for AD.

### 2.10 Publication bias test

We did not examine publication bias and small study effects because of the accumulated number of eligible studies for individual comparison were not more than 10.^39^

## 3 RESULTS

### 3.1 Identification and selection of study

**FIGURE 1:**
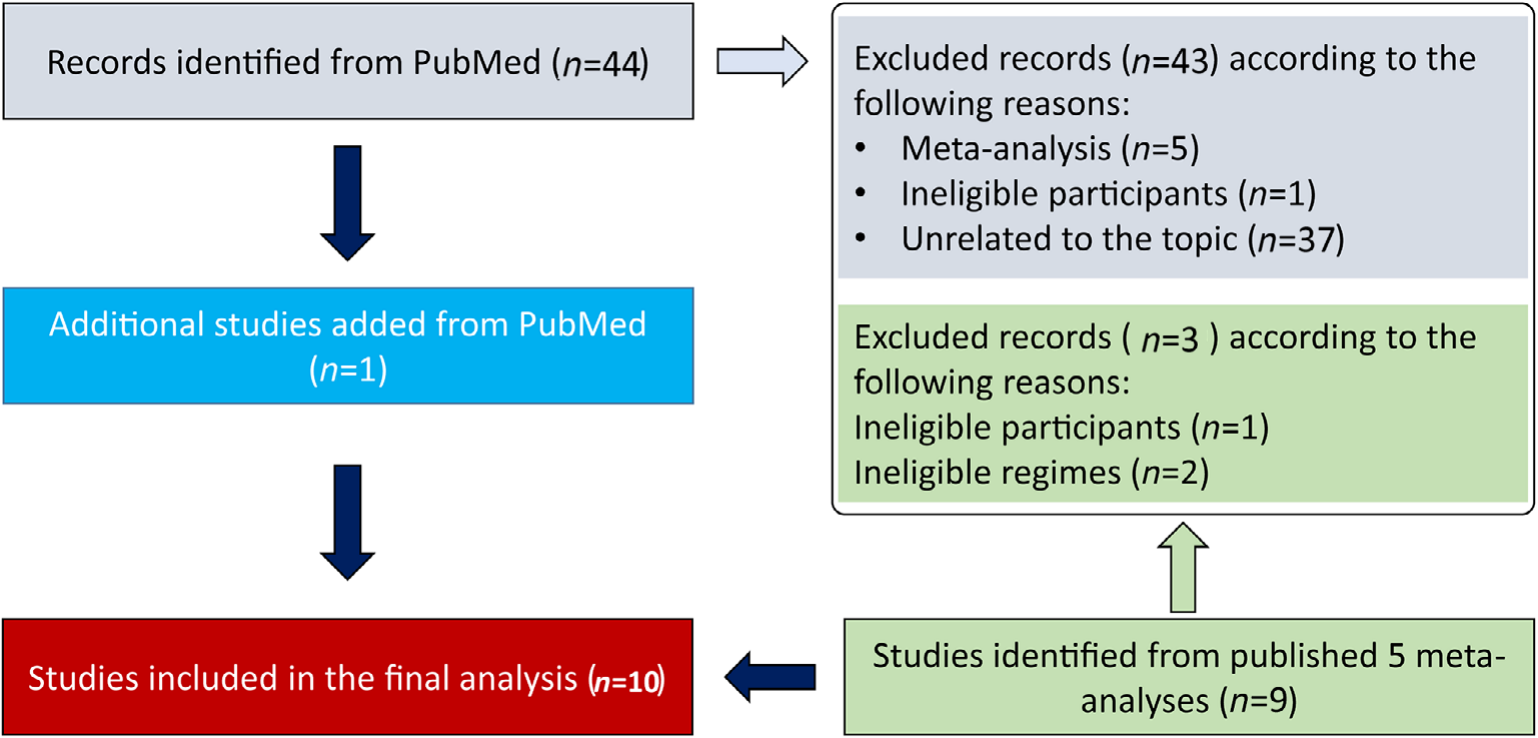
Flow chart of searching and selecting eligible studies

We obtained 44 unique records from PubMed, and included 1 eligible study in the final analysis after excluding 43 ineligible publications due to (a) meta‐analysis (*n* = 5), (b) ineligible participant (*n* = 1), and (c) unrelated to topic (*n* = 37). On the other hand, we obtained 12 potentially eligible publications from published meta‐analyzes, and 9 studies were considered to meet our selection criteria after excluding 3 ineligible studies according to 2 reasons including (a) ineligible participant (*n* = 1) and (b) ineligible treatment regimens (*n* = 2). Finally, a total of 10 studies (two publication^40^, ^41^ includes two studies, respectively)^40^, ^41^, ^42^, ^43^, ^44^, ^45^, ^46^, ^47^, ^48^, ^49^ were included in this network meta‐analysis. The process of identification and selection of studies was depicted in Figure 1.

### 3.2 Basic characteristics of eligible studies and participants

Comparison of abrocitinib and placebo was identified in four studies,^42^, ^43^, ^47^, ^49^ comparison of baricitinib and placebo was identified in three studies,^40^, ^48^ and comparison of upadacitinib and placebo was identified in two studies^41^, ^44^ including three reports. All studies reported efficacy and safety outcomes of interesting. Six publications^40^, ^41^, ^42^, ^47^, ^48^, ^49^ including 8 studies were phase III design, two publications were phase IIb design.^43^, ^44^ All patients enrolled in all eligible studies were confirmed to have moderate to severe AD according to American academy of dermatology (AAD) guideline, Japanese dermatological association (JDA) guideline, or Hanifin and Rajka criteria. The characteristics of eligible studies and participants were summarized in Tables 1 and 2, respectively.

**TABLE 1:** Characteristics of included studies

Study	Phase	Agent	Details of JAK	Mechanism of inhibition	Endpoint	Efficacy outcomes	Safety outcomes
Gooderham et al.^43^	Phase IIb	abrocitinib	10 mg, 30 mg, 100 mg, 200 mg orally once daily	JAK1	12 weeks	IGA, EASI	TEAEs
Silverberg^47^	Phase III	abrocitinib	100 mg and 200 mg orally once daily	JAK1	12 weeks	IGA, EASI	TEAEs
Simpson 2020a^40^	Phase III	abrocitinib	100 mg and 200 mg orally once daily	JAK1	12 weeks	IGA, EASI	TEAEs
Bieber^42^	Phase III	abrocitinib	100 mg and 200 mg orally once daily	JAK1	12 weeks	IGA, EASI	TEAEs
Simpson 2020b^40^	Phase III	baricitinib	1 mg, 2 mg, 4 mg orally once daily	JAK1, JAK2	16 weeks	IGA, EASI	TEAEs
Simpson 2020c^40^	Phase III	baricitinib	1 mg, 2 mg, 4 mg orally once daily	JAK1, JAK2	16 weeks	IGA, EASI	TEAEs
Simpson 2021^48^	Phase III	baricitinib	1 mg and 2 mg orally once daily	JAK1, JAK2	16 weeks	IGA, EASI	TEAEs
Guttman‐Yassky et al.^44^	Phase IIb	upadacitinib	7.5 mg, 15 mg, 30 mg orally once daily	JAK1	16 weeks	IGA, EASI	TEAEs
Guttman‐Yassky et al. 2021a^41^	Phase III	upadacitinib	15 mg and 30 mg orally once daily	JAK1	16 weeks	IGA, EASI	TEAEs
Guttman‐Yassky et al. 2021b^41^	Phase III	upadacitinib	15 mg and 30 mg orally once daily	JAK1	16 weeks	IGA, EASI	TEAEs

Abbreviations: EASI, eczema area and severity index; IGA, investigator global assessment; TEAEs, treatment‐emergent adverse events.

**TABLE 2:** Basic characteristics of participants in eligible studies

Study	Sample size (male)	Mean age, years	Definition of AD	Agent	Dosages	IGA	EASI	TEAEs
Gooderham et al.^43^	211 (102) vs 56 (21)	40.4 vs 42.6	AAD guideline	abrocitinib	100 mg	16/56	22/56	54/56
					200 mg	21/55	31/55	52/55
Silverberg^47^	313 (182) vs 78 (47)	35.5 vs 33.4	Hanifin and Rajka criteria	abrocitinib	100 mg	44/158	69/158	99/158
					200 mg	59/155	94/155	102/155
Simpson 2020a^40^	310 (171) vs 77 (49)	32.8 vs 31.5	Hanifin and Rajka criteria	abrocitinib	100 mg	37/156	62/156	82/156
					200 mg	64/154	96/154	83/154
Bieber^42^	464 (224) vs 131 (77)	38.1 vs 37.4	AAD guideline	abrocitinib	100 mg	86/238	138/238	65/238
					200 mg	106/226	154/226	88/226
Simpson 2020b^40^	375 (243) vs 249 (148)	35.3 vs 35.0	AAD guideline	baricitinib	1 mg	15/127	22/157	67/127
					2 mg	14/123	23/123	72/123
					4 mg	21/125	31/125	73/125
Simpson 2020c^40^	371 (277) vs 244 (154)	34.3 vs 35.0	AAD guideline	baricitinib	1 mg	11/125	16/125	67/125
					2 mg	13/123	22/123	72/123
					4 mg	17/123	26/123	67/123
Simpson 2021^48^	293 (145) vs 147 (80)	40.0 vs 39.0	AAD guideline	baricitinib	1 mg	19/146	19/146	79/146
					2 mg	36/146	44/146	74/146
Guttman‐Yassky et al.^44^	126 (80) vs 41 (24)	40.0 vs 39.9	Hanifin and Rajka criteria	upadacitinib	15 mg	13/42	23/42	32/42
					30 mg	21/42	28/42	33/42
Guttman‐Yassky et al. 2021a^41^	566 (312) vs 281 (144)	33.9 vs 34.4	Hanifin and Rajka criteria	upadacitinib	15 mg	135/281	196/281	176/281
					30 mg	177/285	227/285	209/285
Guttman‐Yassky et al. 2021b^41^	558 (317) vs 278 (154)	33.7 vs 33.4	Hanifin and Rajka criteria	upadacitinib	15 mg	107/276	166/276	166/276
					30 mg	147/282	206/282	173/282

Abbreviations: AD, atopic dermatitis; AAD, American academy of dermatology; EASI, eczema area and severity index; IGA, investigator global assessment; JDA, Japanese dermatological association; TEAEs, treatment‐emergent adverse events.

### 3.3 Methodological quality

According to the Cochrane risk of bias assessment tool, the overall methodological quality was determined as moderate level in four studies^40^, ^42^, ^48^(one publication^40^ includes two studies) and high level in six studies^41^, ^43^, ^44^, ^47^, ^49^ (one publication^41^ includes two studies). Details of risk of bias of each study were summarized in Table 3.

**TABLE 3:** Risk of bias summary of eligible studies

Study	Q1	Q2	Q3	Q4	Q5	Q6	Q7	Overall level
Gooderham et al.^43^	L	L	L	L	L	L	L	High
Silverberg^47^	L	L	L	L	L	L	L	High
Simpson 2020a^40^	L	L	L	L	L	L	L	High
Bieber^42^	U	U	L	L	L	L	L	Moderate
Simpson 2020b^40^	L	L	U	U	L	L	L	Moderate
Simpson 2020c^40^	L	L	U	U	L	L	L	Moderate
Simpson 2021^48^	L	L	U	U	L	L	L	Moderate
Guttman‐Yassky et al.^44^	L	L	L	L	L	L	L	High
Guttman‐Yassky et al. 2021a^41^	L	L	L	L	L	L	L	High
Guttman‐Yassky et al. 2021b^41^	L	L	L	L	L	L	L	High

*Note*: Q1 to Q7 represents random sequence generation, allocation concealment, blinding of personnel and participants, blinding of outcome assessment, incomplete data, selective reporting, and other sources, respectively. L, U, and H indicates low, unclear, and high risk, respectively.

### 3.4 Meta‐analysis of IGA response

**FIGURE 2:**
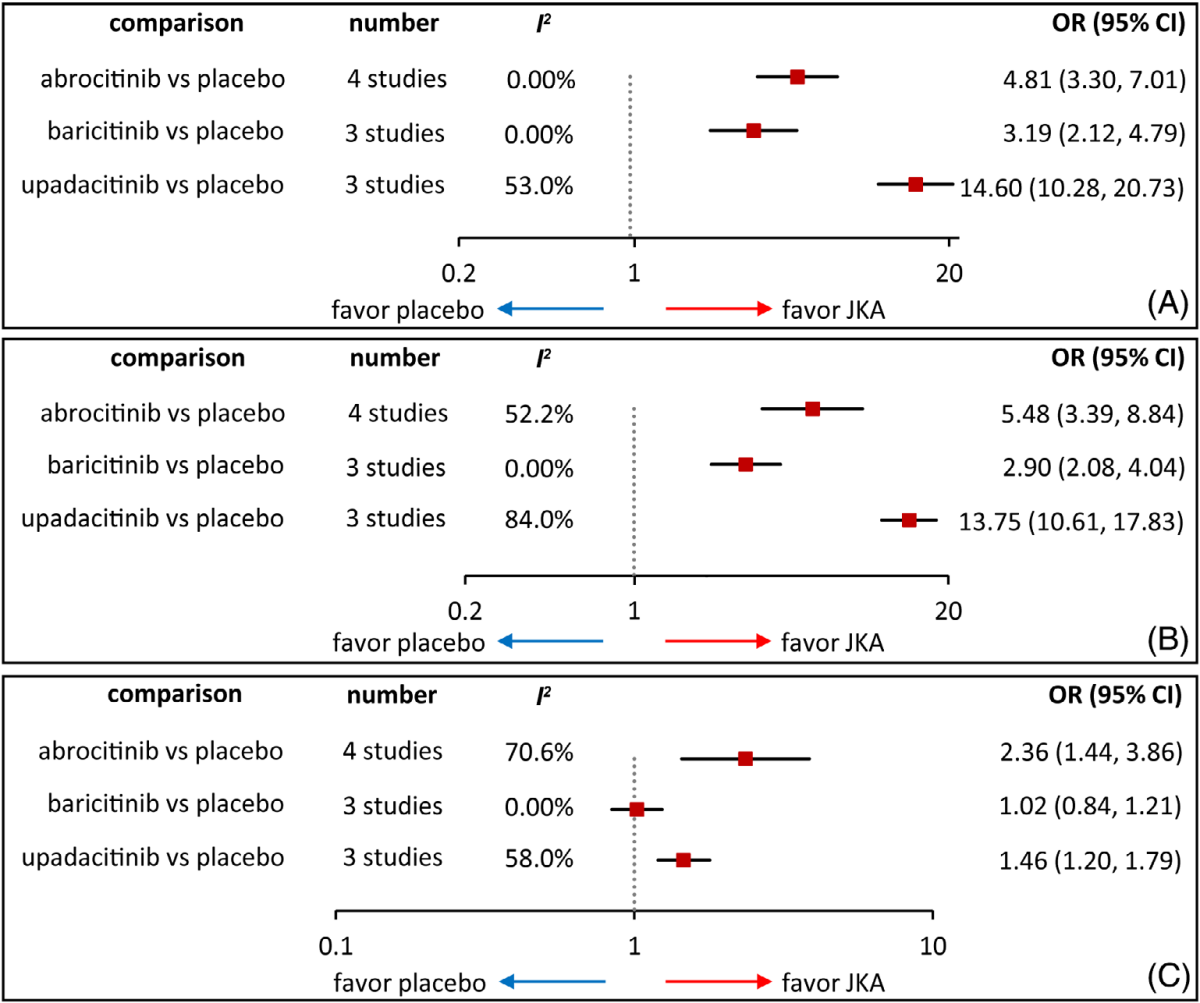
Pair‐wise meta‐analysis of IGA (A), EASI (B), and TEAEs (C). Gray dashed line indicates no statistical significance. Red square represents a point estimate, and black horizontal line represents 95% confidence interval.

**FIGURE 3:**
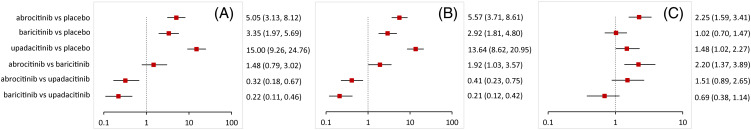
Network meta‐analysis of IGA (A), EASI (B), and TEAEs (C). Gray dashed line indicates no statistical significance. Red square represents a point estimate, and black horizontal line represents 95% credible interval.

All included studies reported IGA response, and three JAK inhibitors were all associated with improved IGA response compared with placebo (Figure 2A), which were consistent with results of network meta‐analyzes (Figure 3A). Moreover, network meta‐analysis suggested that upadacitinib had significantly higher IGA response than abrocitinib and baricitinib (Figure 3A). Furthermore, network meta‐analysis of various doses regimes suggested upadacitinib 30 mg was superior to all other regimens, upadacitinib 15 mg was better than abrocitinib and baricitinib in different dosages apart from abrocitinib 200 mg which was superior to abrocitinib 100 mg, baricitinib 1 mg and baricitinib 2 mg (Table S2).

### 3.5 Meta‐analysis of EASI response

EASI response was reported by all eligible studies. Three JAK inhibitors were found to be better than placebo for EASI response (Figure 2B), which was supported by network meta‐analyzes (Figure 3B). Moreover, upadacitinib was superior to abrocitinib and baricitinib as well as abrocitinib was better than baricitinib (Figure 3B). Furthermore, network meta‐analysis of various doses regimes suggested upadacitinib 30 mg was superior to all other regimens, upadacitinib 15 mg was better than the remaining regimes apart from abrocitinib 200 mg which was superior to abrocitinib 100 mg and baricitinib with different dosages (Table S2).

### 3.6 Meta‐analysis of TEAEs

Direct meta‐analysis suggested, as shown in Figure 2C, an increased incidence of TEAEs in patients treated by abrocitinib (OR 2.25, 95% CI 1.59 to 3.41) and upadacitinib (OR, 1.48, 95% CI 1.02 to 2.27) compared with placebo, which was confirmed in network meta‐analysis (Figure 3C). However, subgroup network meta‐analysis revealed that upadacitinib 30 mg significantly increased the incidence of TEAEs (OR 6.71, 95% CrI 1.48 to 30.83, **Table** **S2**). No statistical difference was detected for other comparisons.

## 4 DISCUSSION

AD is one of the serious global problems,^24^ which affects approximately 15%–30% of children and 10% of adults in high‐income countries.^50^ AD has a major impact on physical and psychological wellbeing,^5^ and eventually will impair health‐related quality of life (QoL)^6^ and increase healthcare costs.^9^ JAK inhibitors have been utilized to treat AD in clinical practice, and several clinical trials^21^, ^22^ and subsequent meta‐analyzes^23^, ^24^, ^25^ have consistently established its efficacy. Different JAK inhibitors were theoretically speculated to have different treatment efficacy for AD, however studies directly comparing different JAK inhibitors with each other in moderate‐to‐severe AD are not available. We conduct this network meta‐analysis to examine comparative data of the most common three oral JAK inhibitors including abrocitinib, baricitinib and upadacitinib simultaneously, and results suggest that upadacitinib 30 mg achieves higher IGA and EASI response compared with other regimens. Moreover, upadacitinib 15 mg also significantly increased IGA or EASI response compared with abrocitinib and baricitinib. However, it is noted that upadacitinib 30 mg caused more TEAEs.

Up to now, three direct meta‐analyzes^51^ exploring role of JAK inhibitors for the treatment of AD, and all consistently suggested that JAK inhibitors were promising treatment option for AD. It must be noted that these meta‐analyzes simultaneously incorporated monotherapy and combination therapy^51^, ^52^ into an individual analysis without separate analyzes according to types of regimes. Meanwhile, adult and pediatric patients were also simultaneously considered,^53^ without sensitivity analysis based on populations. In our network meta‐analysis, only adult and adolescent AD receiving monotherapy are considered to be eligible, and thus more reliable and robust results are obtained compared to previous meta‐analyzes. Moreover, a meta‐analysis also investigated the role of abrocitinib in the treatment of AD, and suggested that dose regimens of 200 mg and 100 mg seemed to have similar benefits,^30^ which are consistent with our findings. However, our network meta‐analysis further determines that dose regime of 200 mg is superior to the regime of 100 mg.

In 2021, one network meta‐analysis has been conducted to investigate the comparative efficacy and safety of systemic therapies used in moderate‐to‐severe AD.^54^ In this network meta‐analysis, authors included upadacitinib 30 mg had the numerically highest efficacy, followed by abrocitinib 200 mg and upadacitinib 15 mg, and reported that similar findings were observed for IGA response. In our network meta‐analysis, however, we find that upadacitinib 30 mg was associated with increased IGA and EASI response compared with all other regimens, and upadacitinib 15 mg was also superior to other regimens except for abrocitinib 200 mg in terms of IGA and EASI response. It must be noted that only three studies for abrocitinib, one study for baricitinib and one study for upadacitinib were identified for monotherapy treatments, however our network meta‐analysis included more studies for these two efficacy outcomes.

The administration of JAK inhibitors was associated with an elevated risk of TEAEs. Unfortunately, a previous meta‐analysis^24^ did not detect statistical difference between JAK inhibitors and placebo for any AEs. Certainly, TEAEs were not separately investigated in this meta‐analysis. However, another meta‐analysis performed by Tsai and colleagues determined more TEAEs among patients received JAK inhibitors (risk ratio (RR) 1.14, 95% CI 1.02 to 1.28).^25^ Network meta‐analysis performed by Silverberg and colleagues suggested no statistical difference between abrocitinib (regardless of doses) and placebo. In our network meta‐analysis, abrocitinib and upadacitinib regardless of doses were found to caused more TEAEs than placebo, and abrocitinib is associated with increased incidence of TEAEs when compared to baricitinib. However, further network meta‐analysis based on dosages revealed that only upadacitinib 30 mg significantly increased the incidence of TEAEs.

Our network meta‐analysis has several strengths. First, we only considered RCTs investigating the role of monotherapy treatments in adult and adolescent AD patients to reduce bias. Second, we utilized network meta‐analysis of determining the comparative efficacy and safety of the most common three JAK inhibitors, which are not directly compared in a single trial to provide more informative evidence for decision‐making. Third, we selected EASI as one of efficacy outcomes according to a previous meta‐analysis^25^ because EASI has been adequately validated and recommended to evaluate the clinical signs of AD in various clinical settings.^55^, ^56^ More importantly, we also utilized IGA to evaluate treatment efficacy as an alternative to EASI because EASI is complex and time‐consuming in routine practice.^57^

Although strength introduced above enhance the reliability and robustness of our findings, some limitations should be further interpreted. First, we cannot avoid variations of doses in eligible studies. Although we combine multiple arms as an individual arm according to the Cochrane's method, it's inevitable to introduce bias. As a result, we further investigate the efficacy and safety of common doses for AD to reduce the risk of bias. Second, we only evaluate the short‐term efficacy and safety due to availability of data, and thus long‐term efficacy and safety should be further investigated. Third, we cannot separately determine the impact of abrocitinib, baricitinib, and upadacitinib on specified TEAEs such as nasopharyngitis, upper respiratory tract infection, and acne due to limited data available. Fourth, although oral and topical JAK inhibitors were available for the treatment of AD, the current network meta‐analysis only investigated the comparative efficacy and safety of three common oral JAK inhibitors, which might limit the comprehensiveness of our findings.

## 5 CONCLUSION

In conclusion, results of this network meta‐analysis highlight that efficacy of JAK1 inhibitors including abrocitinib, baricitinib, and upadacitinib is consistently higher than those of placebo in moderate‐to‐severe AD. Meanwhile, upadacitinib 30 mg should be preferentially considered for AD because it achieves higher IGA and EASI response compared with all other regimes. However, more efforts should also be made to reduce the risk of causing TEAEs.

## AUTHOR CONTRIBUTIONS

*Substantially contributed to conception or design*: Huiying Wan. *Contributed to acquisition, analysis, or interpretation of data*: Haiping Jia. *Drafted the manuscript for important content*: Tian Xia. *Critically revised the manuscript for important intellectual content*: Dingding Zhang. *Gave final approval*: All authors.

## FUNDING INFORMATION

The current study was supported by grants from the Science and Technology Project of Health Commission of Sichuan Province (21PJ088), the Department of Science and Technology of Sichuan Province (Grant No. 20YFS0435), the Health Care of Sichuan Provincial cadres (2020–227), Science & Technology Bureau of Chengdu (YF05‐00198‐SN and YF05‐00060‐SN) and the Sichuan Academy Medical Science & Sichuan Provincial People Hospital (2018LY01).

## CONFLICT OF INTEREST

The authors declare that they have no conflict of interest.

## Supporting information


**Table S1:** Search strategy of PubMed
**Table S2.** Pooled results from network meta‐analysis after conducting subgroup analysis according to the most common doses.

## Data Availability

The data that support the findings of this study are available from the corresponding author upon reasonable request.
