# Supplementary material for: Comparative efficacy and safety of abrocitinib, baricitinib, and upadacitinib for moderate‐to‐severe atopic dermatitis: A network meta‐analysis
Source: Dermatol Ther. 2022 Jul 27;35(9):e15636. doi: 10.1111/dth.15636 (PMC9541568; doi:10.1111/dth.15636)
Supplement: Supplementary file 1 — Table S1: Search strategy of PubMed Table S2. Pooled results from network meta‐analysis after conducting subgroup analysis according to the most common doses. [file DTH-35-e15636-s001.docx]

***Supplementary Material***

**Supplementary Tables**

Table S1. Search strategy of PubMed

| number | Query |
| --- | --- |
| 12 | (("Dermatitis, Atopic"[Mesh]) OR ((((((((Atopic Dermatitides[Title/Abstract]) OR (Atopic Dermatitis[Title/Abstract])) OR (Atopic Neurodermatitides[Title/Abstract])) OR (Atopic Neurodermatitis[Title/Abstract])) OR (Disseminated Neurodermatitides[Title/Abstract])) OR (Disseminated Neurodermatitis[Title/Abstract])) OR (Atopic Eczema[Title/Abstract])) OR (Infantile Eczema[Title/Abstract]))) AND (((("Janus Kinase Inhibitors"[Mesh]) OR "Janus Kinase Inhibitors" [Pharmacological Action]) OR ((((JAK Inhibitors[Title/Abstract]) OR (Janus Kinase Inhibitor[Title/Abstract])) OR (JAK Inhibitor[Title/Abstract])) OR (JAK[Title/Abstract]))) OR (((("abrocitinib" [Supplementary Concept]) OR "baricitinib" [Supplementary Concept]) OR " upadacitinib" [Supplementary Concept]) OR (((abrocitinib[Title/Abstract]) OR (baricitinib[Title/Abstract])) OR (upadacitinib [Title/Abstract])))) from February to July 202. |
| 11 | (("Dermatitis, Atopic"[Mesh]) OR ((((((((Atopic Dermatitides[Title/Abstract]) OR (Atopic Dermatitis[Title/Abstract])) OR (Atopic Neurodermatitides[Title/Abstract])) OR (Atopic Neurodermatitis[Title/Abstract])) OR (Disseminated Neurodermatitides[Title/Abstract])) OR (Disseminated Neurodermatitis[Title/Abstract])) OR (Atopic Eczema[Title/Abstract])) OR (Infantile Eczema[Title/Abstract]))) AND (((("Janus Kinase Inhibitors"[Mesh]) OR "Janus Kinase Inhibitors" [Pharmacological Action]) OR ((((JAK Inhibitors[Title/Abstract]) OR (Janus Kinase Inhibitor[Title/Abstract])) OR (JAK Inhibitor[Title/Abstract])) OR (JAK[Title/Abstract]))) OR (((("abrocitinib" [Supplementary Concept]) OR "baricitinib" [Supplementary Concept]) OR " upadacitinib" [Supplementary Concept]) OR (((abrocitinib[Title/Abstract]) OR (baricitinib[Title/Abstract])) OR (upadacitinib[Title/Abstract])))) |
| 10 | ((("Janus Kinase Inhibitors"[Mesh]) OR "Janus Kinase Inhibitors" [Pharmacological Action]) OR ((((JAK Inhibitors[Title/Abstract]) OR (Janus Kinase Inhibitor[Title/Abstract])) OR (JAK Inhibitor[Title/Abstract])) OR (JAK[Title/Abstract]))) OR (((("abrocitinib" [Supplementary Concept]) OR "baricitinib" [Supplementary Concept]) OR "upadacitinib" [Supplementary Concept]) OR (((abrocitinib[Title/Abstract]) OR (baricitinib[Title/Abstract])) OR (upadacitinib[Title/Abstract]))) |
| 9 | ((("abrocitinib" [Supplementary Concept]) OR "baricitinib" [Supplementary Concept]) OR " upadacitinib" [Supplementary Concept]) OR (((abrocitinib[Title/Abstract]) OR (baricitinib[Title/Abstract])) OR (upadacitinib[Title/Abstract])) |
| 8 | ((abrocitinib[Title/Abstract]) OR (baricitinib[Title/Abstract])) OR (upadacitinib[Title/Abstract]) |
| 7 | (("abrocitinib" [Supplementary Concept]) OR "baricitinib" [Supplementary Concept]) OR " upadacitinib" [Supplementary Concept] |
| 6 | (("Janus Kinase Inhibitors"[Mesh]) OR "Janus Kinase Inhibitors" [Pharmacological Action]) OR ((((JAK Inhibitors[Title/Abstract]) OR (Janus Kinase Inhibitor[Title/Abstract])) OR (JAK Inhibitor[Title/Abstract])) OR (JAK[Title/Abstract])) |
| 5 | (((JAK Inhibitors[Title/Abstract]) OR (Janus Kinase Inhibitor[Title/Abstract])) OR (JAK Inhibitor[Title/Abstract])) OR (JAK[Title/Abstract]) |
| 4 | ("Janus Kinase Inhibitors"[Mesh]) OR "Janus Kinase Inhibitors" [Pharmacological Action] |
| 3 | ("Dermatitis, Atopic"[Mesh]) OR ((((((((Atopic Dermatitides[Title/Abstract]) OR (Atopic Dermatitis[Title/Abstract])) OR (Atopic Neurodermatitides[Title/Abstract])) OR (Atopic Neurodermatitis[Title/Abstract])) OR (Disseminated Neurodermatitides[Title/Abstract])) OR (Disseminated Neurodermatitis[Title/Abstract])) OR (Atopic Eczema[Title/Abstract])) OR (Infantile Eczema[Title/Abstract])) |
| 2 | (((((((Atopic Dermatitides[Title/Abstract]) OR (Atopic Dermatitis[Title/Abstract])) OR (Atopic Neurodermatitides[Title/Abstract])) OR (Atopic Neurodermatitis[Title/Abstract])) OR (Disseminated Neurodermatitides[Title/Abstract])) OR (Disseminated Neurodermatitis[Title/Abstract])) OR (Atopic Eczema[Title/Abstract])) OR (Infantile Eczema[Title/Abstract]) |
| 1 | "Dermatitis, Atopic"[Mesh] |

**Table S2.** Pooled results from network meta-analysis after conducting subgroup analysis according to the most common doses.

| Treatment | Odds ratio [95% CrI] | | | | | | |
| --- | --- | --- | --- | --- | --- | --- | --- |
|  | placebo | abrocitinib 100 mg | abrocitinib 200 mg | baricitinib 1 mg | baricitinib 2 mg | baricitinib 4 mg | upadacitinib 15 mg |
| IGA response | | | | | | | |
| abrocitinib 100 mg | **4.02 (2.58, 6.21)** |  |  |  |  |  |  |
| abrocitinib 200 mg | **7.13 (4.50, 10.96)** | **1.76 (1.34, 2.36)** |  |  |  |  |  |
| baricitinib 1 mg | **2.33 (1.54, 3.81)** | 0.59 (0.31. 1.14) | **0.34 (0.18, 0.65)** |  |  |  |  |
| baricitinib 2 mg | **3.50 (2.24, 5.66)** | 0.88 (0.45, 1.69) | **0.49 (0.25, 0.98)** | 1.51 (0.95, 2.28) |  |  |  |
| baricitinib 4 mg | **4.37 (2.57, 7.23)** | 1.09 (0.53, 2.15) | 0.61 (0.31, 1.21) | **1.82 (1.08, 3.03)** | 1.21 (0.73, 1.97) |  |  |
| upadacitinib 15 mg | **11.42 (7.44, 17.51)** | **2.85 (1.55, 5.11)** | 1.64 (0.87, 2.90) | **4.93 (2.51, 8.74)** | **3.28 (1.71, 6.01)** | **2.63 (1.35, 5.08)** |  |
| upadacitinib 30 mg | **20.20 (13.20, 31.07)** | **5.02 (2.75, 9.34)** | **2.84 (1.58, 5.35)** | **8.72 (4.36, 16.03)** | **5.90 (3.10, 10.90)** | **4.68 (2.47, 8.99)** | **1.76 (1.33, 2.45)** |
| EASI response | | | | | | | |
| abrocitinib 100 mg | **4.52 (3.16, 6.44)** |  |  |  |  |  |  |
| abrocitinib 200 mg | **8.80 (6.19, 12.86)** | **1.94 (1.49, 2.59)** |  |  |  |  |  |
| baricitinib 1 mg | **1.97 (1.26, 3.08)** | **0.44 (0.24, 0.77)** | **0.23 (0.12, 0.39)** |  |  |  |  |
| baricitinib 2 mg | **3.41 (2.28, 5.28)** | 0.75 (0.44, 1.34) | **0.39 (0.22, 0.68)** | **1.74 (1.15, 3.01)** |  |  |  |
| baricitinib 4 mg | **3.96 (2.47, 6.14)** | 0.86 (0.48, 1.57) | **0.45 (.25, 0.79)** | **1.98 (1.24, 3.38)** | 1.13 (0.73, 1.76) |  |  |
| upadacitinib 15 mg | **11.01 (7.82, 15.47)** | **2.38 (1.51, 4.09)** | 1.24 (0.76, 2.02) | **5.59 (3.21, 9.90)** | **3.18 (1.87, 5.42)** | **2.78 (1.61, 4.88)** |  |
| upadacitinib 30 mg | **19.30 (13.32, 27.04)** | **4.21 (2.59, 6.99)** | **2.19 (1.32, 3.54)** | **9.75 (5.50, 17.53)** | **5.56 (3.19, 9.57)** | **4.91 (2.75, 8.68)** | **1.75 (1.27, 2.37)** |
| TEAEs | | | | | | | |
| abrocitinib 100 mg | 3.43 (0.74, 16.26) |  |  |  |  |  |  |
| abrocitinib 200 mg | 3.33 (0.74, 16.54) | 0.98 (0.20, 4.64) |  |  |  |  |  |
| baricitinib 1 mg | 1.01 (0.24, 4.31) | 0.29 (0.03, 2.39) | 0.30 (0.04, 2.50) |  |  |  |  |
| baricitinib 2 mg | 1.12 (0.25, 4.99) | 0.33 (0.04, 2.61) | 0.34 (0.04, 2.73) | 1.10 (0.25, 4.98) |  |  |  |
| baricitinib 4 mg | 1.07 (0.20, 5.99) | 0.31 (0.03, 3.09) | 0.32 (0.03, 3.09) | 1.06 (0.19, 6.06) | 0.96 (0.17, 5.28) |  |  |
| upadacitinib 15 mg | 4.89 (0.49, 48.84) | 1.55 (0.17, 13.03) | 1.56 (0.18, 13.53) | 5.12 (0.61, 44.79) | 4.72 (0.57, 10.43) | 4.89 (0.49, 48.84) |  |
| upadacitinib 30 mg | **6.71 (1.48, 30.83)** | 1.96 (0.22, 16.17) | 1.98 (0.24, 16.76) | 6.62 (0.81, 59.86) | 6.01 (0.75, 51.38) | 6.25 (0.61, 61.12) | 1.28 (0.28, 5.65) |
